# Diminished Late Gestation Placental Volume in Fetal Heart Disease and Implications for Birth Anthropometrics

**DOI:** 10.3390/jcdd13060236

**Published:** 2026-05-31

**Authors:** Marin Jacobwitz, Kushal J. Kapse, Julius Ngwa, Josepheen De Asis-Cruz, Yao Wu, Rathinaswamy Govindan, Caitlin McDermott, Mary T. Donofrio, Adre du Plessis, Catherine Limperopoulos, Nickie Andescavage

**Affiliations:** 1 Division of Vascular Neurology, University of Pennsylvania Health System, Philadelphia, PA 19104, USA; jacobwitzm@chop.edu; 2 Developing Brain Institute, Children’s National Hospital, Washington, DC 20010, USA; kjkapse@childrensnational.org (K.J.K.); jngwa@childrensnational.org (J.N.); jocruz@childrensnational.org (J.D.A.-C.); ywu@childrensnational.org (Y.W.); rgovinda@childrensnational.org (R.G.); cmcdermott@childrensnational.org (C.M.); climpero@childrensnational.org (C.L.); 3 Division of Cardiology, Children’s National Hospital, Washington, DC 20010, USA; mdonofri@childrensnational.org; 4 Prenatal Pediatrics Institute, Children’s National Hospital, Washington, DC 20010, USA; adupless@childrensnational.org

**Keywords:** congenital heart disease, placentation, in vivo placenta, placental volume

## Abstract

Background: The primary objective of this study was to compare the in vivo placenta volume across gestation in fetuses with congenital heart disease (CHD) and healthy controls. The second objective was to determine the relationship between placental volume and both CHD characteristics and neonatal birth anthropometrics. Methods: Pregnant women with a fetal diagnosis of CHD and healthy pregnancies were enrolled in a longitudinal observational study at Children’s National Hospital. A total of 451 fetal MRIs were analyzed from 284 pregnant women (112 mothers/182 scans with CHD; 172 controls/261 scans). In vivo placentas were manually segmented to derive volumes and z-scores. Z-scores were computed from placental volume data derived from control participants for weekly GA bins using means and standard deviations. Z-scores were then assigned to the CHD cohort. A linear mixed effects model with random intercepts clustered by subject was applied to examine the associations between placental volumes and CHD characteristics, including comparing placental volumes between groups according to gestational windows. Results: Overall, placental volumes in CHD were not significantly different than placental volumes from controls. However, in infants delivered at term age, CHD placental volume plateaued in the final four weeks of gestation. Smaller in vivo CHD placental volume z-scores were associated with decreased weight at delivery (*p* ≤ 0.0001). Conclusions: This study identifies that in vivo CHD placentas are abnormal in the final four weeks of gestation. Smaller CHD placentas were associated with decreased birth weight, underscoring the importance of placental development in neonatal anthropometrics.

## 1. Introduction

The placenta is responsible for regulating the in utero environment and for maternal and fetal health during pregnancy [1,2,3,4]. Studies have linked ex utero placental pathology findings and placental insufficiency to later childhood and adult-onset diseases, including neurodevelopmental disability, known as placental programming [2,3,5]. In congenital heart disease (CHD), there has been little improvement in early neurodevelopmental outcomes despite improvements in survival over the last 20 years. Although early theories implicated complications of cardiac surgery as the primary driver of neurodevelopmental disability, recent research has identified the importance of in utero development leading to underlying brain vulnerability with postnatally acquired additional risk [6,7,8,9,10]. Currently identified routine clinical risk factors, excluding the placenta, only account for approximately 30% of the variation in neurodevelopmental outcomes after infant cardiac surgery [11].

At term, the placenta is the largest fetal organ, receiving ~40% of fetal cardiac output [12]. In pregnancies with fetal CHD, ex utero studies revealed small placentas with vascular abnormalities [1,13]. Some propose that placental abnormalities and CHD are due to shared abnormal angiogenic mechanisms early in pregnancy [14,15], or that perfusion and oxygenation changes in CHD affect placental development [1,14,16,17]. Others propose that placental anomalies contribute to the incidence of CHD [14]. Although the exact etiology remains unknown, these prior works highlight the important connections between placental and fetal heart development. Furthermore, numerous studies have demonstrated associations between both fetal brain development and postnatal neurodevelopmental outcomes with abnormal placental pathology in CHD [12,18,19,20,21]. Although it has been consistently shown that placental pathology in CHD is abnormal, there have been mixed reports on when and if CHD placentas are smaller in gestation compared with healthy controls [22,23]. Adding to the complexity of understanding placental growth, it is notoriously difficult to study this critical organ, given its inaccessibility in utero. Ultrasonography is the primary imaging tool utilized in clinical obstetrics for fetal screening and follow-up guidance, yet it provides a limited evaluation of placental function and health [24,25]. Increasingly, in vivo placental magnetic resonance imaging (MRI) has been shown to provide safe and unprecedented access for evaluation of the placenta throughout pregnancy in both healthy and high-risk conditions [23,26,27]. Despite these advances, much less is known about the in vivo placenta in CHD.

The primary objective of this study was to evaluate, in a larger cohort, in vivo placental volume of fetuses with CHD compared to those of healthy control fetuses, with the goal of identifying if and when abnormal placental growth occurs in CHD during gestation. The second objective was to evaluate if particular subpopulations within CHD are at particular risk for adverse placentation.

## 2. Materials and Methods

### 2.1. Patient Population

Subjects with singleton pregnancies greater than 16 weeks’ gestation were enrolled in a longitudinal, prospective observational study from February 2012 to June 2021 at Children’s National Hospital in Washington, D.C. Control participants were identified from an existing database of prospectively enrolled participants that had no significant pregnancy-related and/or chronic medical conditions, normal prenatal screening and anatomical imaging, and subsequent normal neurodevelopmental testing with identical MR acquisition protocols. Women carrying a fetus with confirmed CHD were recruited from the Prenatal Cardiology Program. Exclusion criteria were extracardiac anomalies, multiple gestation pregnancy, maternal contraindication to MRI, known/suspected congenital infection, and/or documented chromosomal abnormalities based on clinical genetic testing. Fetuses with structural brain MRI abnormalities, fetal demise, or postnatal genetic diagnosis were excluded. The study was approved by the institutional review board at Children’s National Hospital. Written informed consent was obtained and HIPPA compliance maintained for all participants.

### 2.2. Study Design

This was a retrospective analysis of prospectively collected data (described above). Participants were invited to complete up to two serial fetal MRIs, one in the second trimester and one in the third trimester, based on gestational age (GA) at enrollment and participant availability.

### 2.3. Fetal Echocardiography

CHD fetuses underwent fetal anatomic and Doppler echocardiography within 48 h of MRI, all reviewed by a single cardiologist (M.T.D.). Fetal CHD diagnoses were categorized based on heart lesion and primary CHD Class (Class I—two-ventricle CHD without aortic obstruction, Class II—two-ventricle CHD with aortic arch obstruction, Class III—single-ventricle CHD without aortic obstruction, Class IV—single-ventricle CHD with aortic obstruction) [28]. For statistical analyses, transposition of the great arteries (TGA), referred to as Class 0, was analyzed separately given it is hemodynamically dissimilar to other Class I defects. Cardiac lesion type was categorized into five groups based on physiology for analyses. Middle cerebral artery (MCA) and umbilical artery (UA) flow velocities were measured with pulse-wave Doppler; pulsatility indices (PIs) were calculated. The cerebroplacental ratio (CPR) was calculated by dividing the MCA PI by the UA PI [29]. Flow at the aortic isthmus was categorized as forward, reversed, or bidirectional.

### 2.4. Fetal MRI

Studies were performed on a 1.5T Discovery MR450 Scanner (GE Healthcare, Milwaukee, WI, USA) using an 8-channel surface receive coil (USAI, Aurora, OH, USA). Single-shot fast spin-echo (SSFSE) T2-weighted images were performed as follows: fat suppressed with TE 160 ms, TR 1100 ms, FOV 420 × 420 mm, 4 mm slice thickness, 0 mm slice gap, and 40 to 60 consecutive slices for full placental coverage in the axial plane. Interleaved acquisition, including odd and even slices, was performed to avoid slice cross-talk. The methods for the segmentations and extracted volumes were consistent within the two cohorts and for the reliability measures. No contrast or sedation was used.

### 2.5. Post-Processing: Image Segmentation and Volume Calculations

The placenta–uterine interface was identified via change in signal intensity using the adjacent uterine wall for guidance and manually outlined in the axial plane and verified in sagittal and coronal planes using ITK-SNAP software (3.8.0) [30]. The volume was converted from mm^3^ to cm^3^ (Supplemental Figure S1a–d). Placental segmentations were manually performed by one of three trained scientists. Intra-rater and inter-rater reliability were measured via Intraclass Correlation Coefficient based on 75% of the cohort. The inter-rater reliability for placental segmentations was 0.97 (0.95–0.98) and intra-rater reliability was 0.92 (0.81–0.96). Of note, all readers were blinded to subject IDs, previous segmentations, and case–control status to derive inter- and intra-rater reliability measurements. All segmentations were completed on raw volumes, without access/review of previously segmented data. Raw images were selected at random to be re-segmented for reliability measures, inclusive of data from the entire gestational window (20–40 weeks) and placentas with variable implantation sites. The methods for the segmentations and extracted volumes were consistent within the two cohorts and for the reliability measures.

Although there are emerging open-source automatic segmentation tools that have been used with differing success [31], there are substantial challenges unique to placental imaging. Imaging success of the placenta is influenced by the position and shape of the placenta, which is diverse relative to other organs, fetal movement leading to image distortion, and implantation of the placenta into the myometrium leading to difficulties discerning the uterine/placental border. Late gestational age scans and/or higher maternal body mass index result in higher imaging noise, further complicating the contrast between placenta/myometrium. Such placental imaging difficulties highlight the challenges for automated placental segmentation, with manual segmentation minimizing errors and remaining the gold standard.

### 2.6. Demographic and Clinical Data

Clinical and demographic data were collected for each subject. GA was calculated based on the last menstrual period or first-trimester ultrasound.

### 2.7. Statistical Analysis

Descriptive statistics of clinical and demographic characteristics are presented. Student’s t-test analysis for normally distributed continuous variables was performed to assess significant differences in means between the two cohorts. Wilcoxon’s rank-sum test was applied to compare non-normally distributed continuous data. The Chi-square or Fisher’s exact test was used for categorical variables and the Kruskal–Wallis test was used for continuous measures. Boxplots and scatterplots were explored to evaluate differences in placental volume by 4-week GA bins among CHD versus control groups based on the available sample size and distribution of available data (Supplemental Table S1). Demographic variables that differed significantly between CHD and control groups were included in the multivariate regression model. With a steady increase in placental volumes through the gestational period and repeated measures per subject, we employed a linear mixed effects (LME) model with random intercepts clustered by subject to examine the associations between placental volumes and CHD characteristics, as well as compare placental volumes between groups according to gestational windows. As there is no standardized accepted consensus on placental volume or placental weight measures [1] due to the variability in establishing normative values in differing populations, we used the healthy control cohort placental volume measurements to represent normal placental volume.

To explore “relative” placental volume in relation to categorical measures of key clinical characteristics in CHD, and to address the significant variability in placental volumes across the wide gestational range included, z-scores were assigned to the CHD cohort after deriving weekly means and standard deviations from the control cohort (Supplemental Table S1). CHD z-scores were used to perform a more nuanced investigation of CHD characteristics relative to significant deviations in placental volume (i.e., <−1 or >1). A linear mixed effects model was used to compare CHD placental volume z-scores with CHD characteristics. LME model results are presented as least squares means, estimates, standard errors, and *p*-values. Linear regression model results are presented as estimates, standard errors, and *p*-values. Assumptions such as multicollinearity, outliers, and normal distributions of placental volumes were tested in each model. *p*-values < 0.05 were considered statistically significant and false discovery rate assessments were performed for multiple comparisons. Data analysis was conducted by a single statistician (J.N.) using Statistical Analysis System software 9.4 (SAS Institute, Cary, NC, USA) and RStudio 2023.09.1 Build 494.

## 3. Results

### 3.1. Characteristics of CHD and Control Cohorts

A total of 298 pregnant women were enrolled between 16 and 40 weeks’ gestation, 172 healthy controls and 126 with pregnancies complicated by fetal CHD, of whom 166 women had two fetal MRIs, for a total of 464 scans (261 controls and 203 cases with CHD). Of those enrolled, 6 participants (7 scans) from the CHD cohort were excluded (4 due to postnatally confirmed or suspected genetic syndrome, 2 due to in utero fetal demise; 8 participants (14 scans) were excluded due to excessive motion artifact rendering segmentation impossible. The final CHD sample was 112 mothers (182 scans); of the 112 mothers, 70 had two MRIs during their pregnancy. The final control cohort comprised 172 subjects (261 scans); 89 had two MRIs during their pregnancy (Supplemental Figure S2).

Demographic and clinical characteristic comparisons of the control and CHD cohorts are in Table 1. Demographic and clinical characteristics of the CHD cohort are shown in Supplemental Tables S2 and S3.

### 3.2. Placental Volume Comparison Between CHD vs. Control

**Figure 1:**
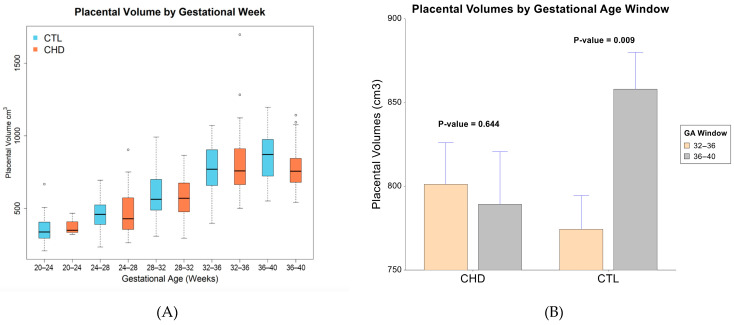
Congenital heart disease (CHD) vs. control (CTL) placental volume by four-week gestational age intervals (**A**) and in the final eight weeks of gestation (**B**).

Placental volumes increased with GA for both the control and CHD cohorts (Supplemental Figure S3). Overall, placental volumes in CHD were similar to control placental volumes after correcting for fetal sex, GA at scan, maternal race, and maternal education level (β = −12.57, SE = 21.36, *p* = 0.557; Supplemental Table S4) with a mean placental volume of 670 cm^3^ in CHD and 671 cm^3^ in controls. However, a detailed comparison of placental volumes between CHD and controls across distinct gestational windows shows a notable difference in placental volume for the CHD cohort in the final four weeks of gestation (Figure 1A). Linear regression models revealed a significant increase in placental volume in the CTL cohort from the 32–36 weeks’ GA window to the 36–40 weeks’ GA window (β = 66.26, SE = 24.9, *p* = 0.009; Figure 1B). However, in the CHD cohort, placental volume plateaued with no significant change in placental volume between the same GA windows (β = −29.7, SE = 63.65, *p* = 0.644; Figure 1B).

### 3.3. Exploratory Analyses of Placental Volume Z-Scores in CHD with Clinical Characteristics

Using the control group placental volumes (N = 264 placentas), normative mean and standard deviations were created for each gestational week and from these, z-scores were then assigned to the CHD placental volumes. Z-scores were used to explore the relative CHD placental volumes with key clinical characteristics and outcomes. First, using a linear mixed effects model, we compared CHD placental volume z-scores accounting for fetal sex, maternal race, maternal education and maternal age at MRI scan to the normal control population mean and did not detect significant differences (Supplemental Table S5). We then categorized CHD placental z-scores into five groups (Table 2) to perform exploratory analyses examining placental volume with CHD characteristics, fetal echocardiogram, and birth outcomes. Univariate exploratory analysis revealed no significant association between placental volume z-score and CHD Class (*p* = 0.350) or CHD lesion type (*p* = 0.202; Table 2).

**Figure 2:**
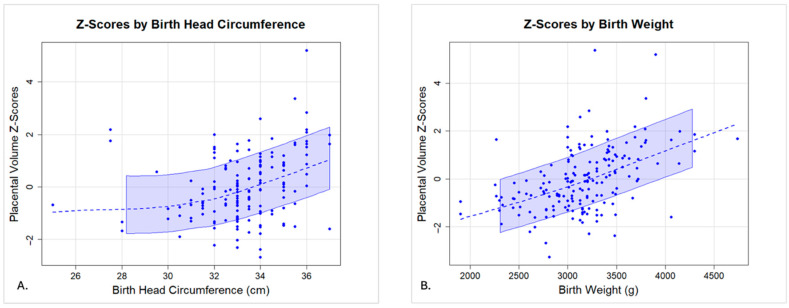
Association between placental volume z-score and birth head circumference (**A**) and birth weight (**B**) in CHD.

Exploratory analyses noted a lower placental volume z-score was associated with smaller head circumference at birth (*p* = 0.02; Table 2; Figure 2A) and lower birth weight (*p* < 0.0001; Table 2; Figure 2B), the latter of which survived multiple corrections.

## 4. Discussion

In this large cohort of fetal CHD, we demonstrate that average placental volume does not differ between CHD and controls in the second half of pregnancy; however, CHD placental volumes demonstrate the absence of an expected increase at the end of the third trimester. Our large sample size has allowed for a more detailed comparison of placental volumes in discrete gestational windows. The notable lack of an expected increase in placental volume between 36 and 40 weeks’ gestation in CHD is contrasted by a period of ongoing and significant increase in placental volumes and fetal growth in healthy controls, and likely represents a period of particular vulnerability for fetuses with CHD. Moreover exploratory analyses of these in vivo measures of placental volumes in CHD were associated with smaller head circumferences at birth and, more significantly, lower birth weight, highlighting the important relationship between in vivo placental development and fetal growth.

Placental plasticity, known as the period during gestation when placental growth occurs in response to the nutrient and metabolic demands placed upon it, is present for a constrained period of time during gestation [32]. However, when this period of placental plasticity has passed, the placenta may no longer adapt as readily to dynamic changes in metabolic demands of the late-gestation fetus [32,33]. This change in placental plasticity may limit the ability of the fetus to tolerate negative influences in the maternal–fetal environment [32]. Indeed, clinical evidence of placental dysfunction often manifests in the second half of the pregnancy when the absolute fetal growth rate per unit placenta is most rapid and outstrips placental capacity [33].

It is important to note that there have been mixed reports on placental weight and volumes in pregnancies complicated by CHD and healthy controls. Despite several in vivo and ex vivo studies by our group and others that have reported no difference in placental volumes in fetal CHD [23,34,35,36], there also are reports of decreased placental growth in CHD [22]. These studies, particularly pathology studies of placental weight, highlight that certain CHD lesions are more likely to result in lower placental weights [1,37,38], contrary to our data demonstrating that placental volumes were independent of CHD lesion type. Variability in both studies of placental volume and weight likely reflect differences in sample size, timing of assessments, heterogeneity of lesion types studied, and unmeasured clinical and environmental factors. Despite these considerations, abnormal placentation in the third trimester in babies born with CHD is not unexpected. Postnatal assessments of placentas for neonates with CHD that reveal smaller placentas also report high rates of vascular abnormalities [1,39]. More specifically, pathology studies of CHD placentas have shown increased rates of maternal vascular malperfusion (MVM) compared with controls [39,40]. Although not well understood, MVM has been theorized to result from abnormal early implantation, including inadequate remodeling of the uterine vasculature and spiral arteries [39,41]. Despite likely origins in early pregnancy, MVM is considered a characteristic finding of chronic placental insufficiency [41,42], as well as intrauterine growth restriction, which often does not manifest until later in gestation [43]. Similarly, chronic oxidative placental stress leads to the accumulation of damaged nuclei in the syncytiotrophoblast towards term gestation in the form of syncytial knots, often causing placental senescence [44], which may be enhanced in the setting of CHD and chronic feto-placental hypoxia. Such disruptions may accumulate over time and only manifest when metabolic demands of the placenta are highest, namely at the end of gestation, as seen in this work. Moreover, the relationship between the absence of an expected placental volumetric increase prenatally in CHD and smaller anthropometric measures at birth may provide further evidence of abnormal placentation [45,46].

Importantly, the lack of an expected increase in placental volumes during the third trimester in fetuses with CHD also coincides with a critical period of accelerated fetal growth and brain development [47,48]. This exponential growth in the brain during the third trimester requires a marked increase in oxygen and nutrient supply from the placenta [47,48]. Previous studies in fetuses with CHD have revealed smaller brain volumes, decreased brain maturation, and abnormal brain metabolism when compared to healthy controls evaluated via in vivo fetal brain MRI and MR spectroscopy [6,7,9]. The field of neuroplacentology has identified a link between abnormal placentation in pregnancies afflicted by CHD and risk of later neurodevelopmental delays [49], with a higher incidence of severe brain injury in infants with CHD and abnormal placental pathology [50], highlighting the importance of understanding the heart–brain–axis. Furthermore, fetuses with CHD and placental dysfunction have longer hospital lengths of stay and higher mortality than fetuses with CHD and healthy placentas [12]. The delayed expansion of placental volume in CHD at 36–40 weeks, along with the association between placental volume and head circumference in newborns with CHD, provide additional evidence that placental volumetric growth restriction may contribute to impaired fetal development in CHD. We posit that the etiology of late-third trimester placental volumetric growth restriction may be the delayed manifestation of early aberrations in placental development, with the exact mechanisms of placental dysfunction in CHD requiring further exploration.

An interlinked placenta–heart axis is a well-established concept with many initial frameworks presuming that the cardiac defect drives the abnormal placenta [51,52] or that the abnormal placenta emerges from a shared ontogeny [52]. Gene expression profiles between first-trimester placenta and heart cell types have revealed commonly expressed genes and pathways known to be associated with both CHD and placenta-related pregnancy complications [52]. More recent work in animal models, however, have also shown the development of CHD secondary to a defective placenta, emphasizing the placenta as a notable source of developmental heart defects [51,52]. Indeed, this work and others suggest that placental pathology is broadly associated with CHD and is not specific to any single condition or lesion type. Collectively, these data suggest that the abnormal placentation may be a primary driver of an adverse in utero environment in CHD, negatively influencing fetal growth and development. Continued work is underway to further investigate this theory.

### Strengths and Limitations

Several strengths of this work are the large sample size and serial imaging available in fetuses with CHD compared to the placentas of healthy control fetuses. Fetal echocardiography and Doppler measurements for the CHD cohort were obtained temporally close to MRI allowing for excellent correlation between echocardiography measurements and placental volumes.

Despite the many strengths of this work, there are limitations that should be acknowledged. First, placental segmentation was done manually, though our inter-rater reliability of placental segmentations was excellent with ICC > 0.95. Second, because CHD is often diagnosed after 20 weeks’ gestation, evaluation of placental volumes and growth prior to 20 weeks’ gestation was not possible. Similarly, based on the diagnosis of CHD and timing of enrollment, not all participants completed two MRIs; though key characteristics between participants were similar, there may have been residual unmeasured bias between participants who completed one versus two prenatal MRI studies. Third, though genetic testing is universally offered to all patients with CHD clinically, prenatal genetic testing was only completed on 51% of the CHD cohort. Although we excluded all fetuses with underlying genetic syndromes based on clinical genetic testing (prenatal and/or postnatal), fetuses with delayed or undiagnosed genetic syndromes may have been inadvertently included. Fourth, although this study included a large sample of control placental MRIs, the number of placentas per GA window for z-score categorization would be optimized with additional subjects and should be considered in future studies. Fifth, despite the use of novel in vivo volumetric placental MRI techniques, this study focused only on structural characteristics of the placenta via volumetric measurements. Though placental structure has been associated with placental insufficiency and growth restriction, it is important to note that placental volume alone is insufficient to measure placental function. Moreover, this study did not include early first trimester measures, which may be insensitive to capture the extent of altered placental development earlier in gestation. Future studies should explore functional placental imaging along with structural imaging throughout gestation, as well as pathologic evaluation after delivery. Sixth, although we attempted to account for covariates that impact the placenta, such as GA at scan, fetal sex, maternal race, maternal education, and maternal age at scan, there may be additional covariates that influence the placenta and in utero environment that were not accounted for in this study, such as pre-pregnancy maternal BMI, among others. Seventh, postnatal data collection in the controls was limited; we were therefore unable to explore associations between placental volumes and birth anthropometrics in the controls. In addition, though infants in this report were delivered at term age, late-third trimester placental volumes in infants at risk for preterm delivery are not represented. Given the associated risks of impaired placental development and premature delivery, additional study of in vivo placental volumes in infants with CHD that are delivered preterm is warranted. Finally, as this study is a retrospective analysis of prospectively collected data, there may be inherent biases associated with this design.

## 5. Conclusions

In conclusion, placental volumes of fetuses with congenital heart disease demonstrate a notable absence of the expected increase in the final four weeks of gestation. Furthermore, lower placental volume z-scores are associated with smaller birth head circumference and birth weight. As the placenta is the primary lifeline for the fetus in utero, improving abnormal placentation may improve placental support of the fetus, in the hope of mitigating perinatal neurologic vulnerability and improving neurodevelopmental outcomes in children with congenital heart disease. Though this work focused on placental volumetric measurements, causality between volumetric and functional placental metrics cannot be inferred. Validation of these findings in prospective studies, as well as expansion of this work to integrate placental function, somatic fetal growth and fetal brain development with both placental structure and pregnancy outcomes, is warranted. In addition to increasing our understanding of the in vivo placenta in congenital heart disease, such studies may help us identify maternal–fetal dyads that may benefit from increased late-third trimester surveillance and personalized peri-partum management.

## Figures and Tables

**Table 1:** CHD versus control cohort comparisons.

Variable	CTL	CHD	*p*-Value
N (%)	(N = 172)	(N = 112)	
Mean ± SD			
Sex of Fetus—Male	89 (52)	74 (66)	0.024
Maternal Race ^+^			<0.001
Asian/Pacific Islander	7 (4)	7 (6)	
Black, not of Hispanic Origin	66 (38)	20 (18)	
Hispanic	18 (10)	16 (14)	
White, not of Hispanic Origin	56 (33)	64 (56)	
Other *	14 (8)	5 (4)	
Unknown	11 (6)	0 (0)	
Maternal Education Level			0.005
High School Graduate or Less	36 (21)	15 (13)	
Any Secondary Education	70 (41)	41 (36)	
Graduate Professional Training	55 (32)	34 (30)	
Unknown	11 (6)	22 (19)	
Maternal Age at Scan (years) ^•^	31.14 ± 6.71	33.53 ± 5.19	0.001
Primiparous			
Yes	46 (27)	34 (30)	0.509
Gravida	2.33 ± 1.35	2.41± 1.65	0.577
Birth Weight (g)	3375 ± 352	3169 ± 460	<0.001
GA at Birth (weeks)	39.52 ± 1.06	38.73 ± 0.99	<0.001
Fetal GA at MRI Scan	30.76 ± 5.61	31.61 ± 4.07	0.068
Fetal GA at MRI Scan 1	27.69 ± 3.43	29.46 ± 3.61	0.004
Fetal GA at MRI Scan 2	35.73 ± 2.23	34.88 ± 2.07	0.023
Placental Volume (cm^3^)	634 ± 239	661 ± 216	0.203
Placental Volume (cm^3^) at Scan 1	437 ± 142	581 ± 205	0.046
Placental Volume (cm^3^) at Scan 2	685 ± 173	784 ± 172	0.286

* American Indian, Alaska Native, Native Hawaiian; ^+^ self-reported; ^•^ per scan (CTL N = 261; CHD N = 182); mean (SD) and Kruskal–Wallis test for continuous measures; count (%) and Fisher’s exact test for categorical measures.

**Table 2:** Exploratory analyses of CHD placental volume z-score categories and key clinical characteristics.

Variable	Z-Score	Z-Score	Z-Score	Z-Score	Z-Score	*p*-Value	FDR *p*-Value
N (%)	</=−1	−1 to −0.5	−0.5–0.5	0.5 to 1	>/=1		
Mean ± SD	N = 45	N = 33	N = 47	N = 19	N = 38		
Maternal Age at Scan *	33.0 ± 5.4	30.6 ± 3.9	35.7 ± 6.0	34.2 ± 4.8	33.5 ± 3.9	0.172	0.469
Sex of Fetus *						0.231	0.469
Male	25 (74)	10 (71)	16 (57)	11 (85)	11 (52)		
Female	9 (26)	4 (29)	12 (43)	2 (15)	10 (48)		
CHD Characteristics							
Primary CHD Class *						0.350	0.525
Class 0	11 (32)	4 (29)	6 (21)	2 (15)	2 (10)		
Class I	5 (15)	5 (36)	10 (36)	5 (38)	4 (19)		
Class II	6 (18)	0 (0)	2 (7)	3 (23)	3 (14)		
Class III	4 (12)	2 (14)	5 (18)	0 (0)	4 (19)		
Class IV	8 (24)	3 (21)	5 (18)	3 (23)	8 (38)		
CHD Lesion *						0.202	0.469
HLHS/Other Functional Single-Ventricle Anomaly	11 (32)	5 (36)	10 (36)	3 (23)	11 (52)		
dTGA (with or without IVS/VSD)	11 (32)	4 (29)	6 (21)	2 (15)	2 (10)		
VSD (with or without IAA/Coarctation)	1 (3)	1 (7)	0 (0)	2 (15)	3 (14)		
TOF (With or without PA	3 (9)	0 (0)	7 (25)	1 (8)	1 (5)		
or MAPCAs)							
Other	8 (24)	4 (29)	5 (18)	5 (38)	4 (19)		
Cardiac Lesion Type *						0.547	0.746
Acyanotic	2 (6)	1 (7)	1 (4)	2 (15)	2 (10)		
Cyanotic	21 (62)	9 (64)	21 (75)	9 (69)	17 (81)		
TGA	11 (32)	4 (29)	6 (21)	2 (15)	2 (10)		
Fetal Echocardiogram							
Ductus Flow *						0.805	0.863
Forward	25 (81)	9 (69)	20 (77)	9 (69)	16 (80)		
Reversed	2 (6)	1 (8)	3 (12)	2 (15)	3 (15)		
Bidirectional	3 (10)	1 (8)	1 (4)	2 (15)	1 (5)		
Isthmus Flow *						0.716	0.853
Forward	20 (65)	10 (77)	21 (81)	9 (69)	10 (50)		
Reversed	8 (26)	3 (23)	4 (15)	3 (23)	8 (40)		
Bidirectional	2 (6)	0 (0)	1 (4)	1 (8)	2 (10)		
Middle Cerebral Artery PI	1.8 ± 0.4	1.8 ± 0.4	1.8 ± 0.4	1.9 ± 0.5	1.8 ± 0.5	0.739	0.853
Umbilical Artery Pulsatility Index	1.2 ± 0.2	1.1 ± 0.3	1.0 ± 0.3	1.2 ± 0.3	1.1 ± 0.3	0.193	0.469
Umbilical Artery Resistivity Index	0.7 ± 0.1	0.7 ± 0.1	0.6 ± 0.1	0.7 ± 0.1	0.7 ± 0.1	0.250	0.469
Cerebroplacental Ratio	1.6 ± 0.4	1.7 ± 0.5	2.1 ± 1.8	1.7 ± 0.4	1.6 ± 0.5	0.302	0.503
Birth Outcomes							
Gestational Age at Birth *	38.6 ± 1.0	38.8 ± 0.9	38.8 ± 1.1	38.7 ± 0.5	38.8 ± 1.2	0.908	0.908
Birth Head Circumference (cm)	32.9 ± 1.7	31.9 ± 2.6	33.7 ± 1.3	33.6 ± 1.6	34.0 ± 2.2	0.020	0.150
Birth Weight (g)	2956 ± 415	2917 ± 354	3169 ± 339	3395 ± 354	3525 ± 535	<0.0001	<0.0001
Birth Length (in) *	48.5 ± 2.1	48.4 ± 4.0	49.5 ± 2.6	50.1 ± 1.0	50.2 ± 4.1	0.247	0.469

Mean (SD) and Kruskal–Wallis test for continuous measures; count (%) and Fisher’s exact test for categorical measures; * variables that do not change between scan 1 and scan 2.

## Data Availability

The data presented in this study are available on request from the corresponding author due to privacy and legal restrictions.

## Supplementary Materials

The following supporting information can be downloaded at: https://www.mdpi.com/article/10.3390/jcdd13060236/s1, Figure S1: Placental MRI Images; Figure S2: Cohort; Figure S3: CHD vs Control Raw Placental Volumes Across Gestation; Table S1: Raw Placental Volume MRI Data from Control Subjects Utilized to Create PV Z-Scores; Table S2: Demographics of CHD Cohort; Table S3: Demographics of CHD Cohort: 1 vs. 2 MRI studies; Table S4: Linear Mixed Effects Model of Association of Placental Volume in CHD vs CTL; Table S5: Linear Mixed Effects Model Demonstrating the Association of CHD Cohort Placental Volume Z-Scores with CTL Population Mean.

## References

[1] Rychik J., Goff D., McKay E., Mott A., Tian Z., Licht D.J., Gaynor J.W. (2018). Characterization of the Placenta in the Newborn with Congenital Heart Disease: Distinctions Based on Type of Cardiac Malformation. Pediatr. Cardiol..

[2] Gluckman P.D., Hanson M.A., Phil D., Cooper C., Thornburg K.L. (2008). Effect of In Utero and Early-Life Conditions on Adult Health and Disease. N. Engl. J. Med..

[3] Burton G.J., Fowden A.L., Thornburg K.L. (2016). Placental Origins of Chronic Disease. Physiol. Rev..

[4] Sandovici I., Hoelle K., Angiolini E., Constância M. (2012). Placental adaptations to the maternal-fetal environment: Implications for fetal growth and developmental programming. Reprod. BioMed Online.

[5] Barker D.J.P., Gelow J., Thornburg K., Osmond C., Kajantie E., Eriksson J.G. (2010). The Early Origins of Chronic Heart Failure: Impaired Placental Growth and Initiation of Insulin Resistance in Childhood. Eur. J. Heart Fail..

[6] Limperopoulos C., Tworetzky W., McElhinney D.B., Newburger J.W., Brown D.W., Robertson R.L., Guizard N., McGrath E., Geva J., Annese D. (2010). Brain volume and metabolism in fetuses with congenital heart disease: Evaluation with quantitative magnetic resonance imaging and spectroscopy. Circulation.

[7] Licht D.J., Shera D.M., Clancy R.R., Wernovsky G., Montenegro L.M., Nicolson S.C., Zimmerman R.A., Spray T.L., Gaynor J.W., Vossough A. (2009). Brain maturation is delayed in infants with complex congenital heart defects. J. Thorac. Cardiovasc. Surg..

[8] Sun L., Macgowan C.K., Sled J.G., Yoo S.J., Manlhiot C., Porayette P., Grosse-Wortmann L., Jaeggi E., McCrindle B.W., Kingdom J. (2015). Reduced fetal cerebral oxygen consumption is associated with smaller brain size in fetuses with congenital heart disease. Circulation.

[9] Miller S.P., McQuillen P.S., Hamrick S., Xu D., Glidden D.V., Charlton N., Karl T., Azakie A., Ferriero D.M., Barkovich A.J. (2007). Abnormal Brain Development in Newborns with Congenital Heart Disease. N. Engl. J. Med..

[10] Licht D.J., Jacobwitz M., Lynch J.M., Ko T., Boorady T., Devarajan M., Heye K.N., Mensah-Brown K., Newland J.J., Schmidt A. (2023). Impaired Maternal-Fetal Environment and Risk for Preoperative Focal White Matter Injury in Neonates with Complex Congenital Heart Disease. J. Am. Heart Assoc..

[11] Gaynor J.W. (2014). The Encephalopathy of Congenital Heart Disease. J. Thorac. Cardiovasc. Surg..

[12] Leon R.L., Mir I.N., Herrera C.L., Sharma K., Spong C.Y., Twickler D.M., Chalak L.F. (2021). Neuroplacentology in Congenital Heart Disease: Placental Connections to Neurodevelopmental Outcomes. Pediatr. Res..

[13] Desmond A., Imany-Shakibai H., Wong D., Kwan L., Satou G., Sklansky M., Afshar Y. (2023). Prenatal Congenital Heart Disease and Placental Phenotypes. JACC Adv..

[14] Courtney J.A., Cnota J.F., Jones H.N. (2018). The role of abnormal placentation in congenital heart disease; Cause, correlate, or consequence?. Front. Physiol..

[15] Binder J., Carta S., Carvalho J.S., Kalafat E., Khalil A., Thilaganathan B. (2020). Evidence for uteroplacental malperfusion in fetuses with major congenital heart defects. PLoS ONE.

[16] Burton G.J., Jauniaux E. (2018). Development of the human placenta and fetal heart: Synergic or independent?. Front. Physiol..

[17] Kaltman J.R., Di H., Tian Z., Rychik J. (2005). Impact of congenital heart disease on cerebrovascular blood flow dynamics in the fetus. Ultrasound Obstet. Gynecol..

[18] Nijman M., van der Meeren L.E., Nikkels P.G., Stegeman R., Breur J.M., Jansen N.J., Ter Heide H., Steenhuis T.J., de Heus R., Bekker M.N. (2024). Placental Pathology Contributes to Impaired Volumetric Brain Development in Neonates With Congenital Heart Disease. J. Am. Heart Assoc..

[19] Segar D.E., Zhang J., Yan K., Reid A., Frommelt M., Cohen S. (2023). The Relationship Between Placental Pathology and Neurodevelopmental Outcomes in Complex Congenital Heart Disease. Pediatr. Cardiol..

[20] Shallie P.D., Naicker T. (2019). The Placenta as a Window to the Brain: A Review on the Role of Placental Markers in Prenatal Programming of Neurodevelopment. Int. J. Dev. Neurosci..

[21] Cohen J.A., Rychik J., Savla J.J. (2021). The Placenta as the Window to Congenital Heart Disease. Curr. Opin. Cardiol..

[22] Jacobwitz M., Kapse K., Ngwa J., De Asis-Cruz J., Wu Y., Donofrio M.T., McDermott C., du Plessis A., Limperopoulos C., Andescavage N. (2025). Placental and Fetal In Utero Growth Among Fetuses With Congenital Heart Disease. JAMA Netw. Open..

[23] Andescavage N., Yarish A., Donofrio M., Bulas D., Evangelou I., Vezina G., McCarter R., Limperopoulos C. (2015). 3-D Volumetric MRI Evaluation of the Placenta in Fetuses with Complex Congenital Heart Disease. Placenta.

[24] Avni R., Neeman M., Garbow J.R. (2015). Functional MRI of the placenta—From rodents to humans. Placenta.

[25] Dahdouh S., Andescavage N., Yewale S., Yarish A., Lanham D., Bulas D., du Plessis A.J., Limperopoulos C. (2018). In vivo placental MRI shape and textural features predict fetal growth restriction and postnatal outcome. J. Magn. Reson. Imaging.

[26] Andescavage N.N., Limperopoulos C. (2021). Placental abnormalities in congenital heart disease. Transl. Pediatr..

[27] Amgalan A., Kapse K., Krishnamurthy D., Andersen N.R., Izem R., Baschat A., Quistorff J., Gimovsky A.C., Ahmadzia H.K., Limperopoulos C. (2022). Measuring intrauterine growth in healthy pregnancies using quantitative magnetic resonance imaging. J. Perinatol..

[28] Clancy R.R., McGaurn S.A., Wernovsky G., Spray T.L., Norwood W.I., Jacobs M.L., Murphy J.D., Gaynor J.W., Goin J.E. (2000). Preoperative risk-of-death prediction model in heart surgery with deep hypothermic circulatory arrest in the neonate. J. Thorac. Cardiovasc. Surg..

[29] Ebbing C., Rasmussen S., Kiserud T. (2007). Middle cerebral artery blood flow velocities and pulsatility index and the cerebroplacental pulsatility ratio: Longitudinal reference ranges and terms for serial measurements. Ultrasound Obstet. Gynecol..

[30] Yushkevich P.A., Piven J., Hazlett H.C., Smith R.G., Ho S., Gee J.C., Gerig G. (2006). User-guided 3D active contour segmentation of anatomical structures: Significantly improved efficiency and reliability. Neuroimage.

[31] Li J., Shi Z., Zhu J., Liu J., Qiu L., Song Y., Wang L., Li Y., Liu Y., Zhang D. (2024). Placenta segmentation in magnetic resonance imaging: Addressing position and shape of uncertainty and blurred placenta boundary. BioMed Signal Process Control.

[32] Thornburg K.L., Marshall N. (2015). The placenta is the center of the chronic disease universe. Am. J. Obstet. Gynecol..

[33] Huppertz B., Peeters L.L.H. (2005). Vascular biology in implantation and placentation. Angiogenesis.

[34] Cromb D., Steinweg J., Aviles Verdera J., van Poppel M.P., Bonthrone A.F., Lloyd D.F., Pushparajah K., Simpson J., Razavi R., Rutherford M. (2025). T2*-Relaxometry MRI to Assess Third Trimester Placental and Fetal Brain Oxygenation and Placental Characteristics in Healthy Fetuses and Fetuses With Congenital Heart Disease. J. Magn. Reson. Imaging.

[35] Josowitz R., Ho D.Y., Shankar S., Mondal A., Zavez A., Linn R.L., Tian Z., Gaynor J.W., Rychik J. (2025). Congenital Heart Disease Fetuses Have Decreased Mid-Gestational Placental Flow, Placental Malperfusion Defects, and Impaired Growth. JACC Adv..

[36] Cromb D., Slator P.J., Hall M., Price A., Alexander D.C., Counsell S.J., Hutter J. (2024). Advanced magnetic resonance imaging detects altered placental development in pregnancies affected by congenital heart disease. Sci. Rep..

[37] Snoep M.C., Aliasi M., van der Meeren L.E., Jongbloed M.R.M., DeRuiter M.C., Haak M.C. (2021). Placenta morphology and biomarkers in pregnancies with congenital heart disease—A systematic review: Review on congenital heart disease and placenta morphology. Placenta.

[38] Matthiesen N.B., Henriksen T.B., Agergaard P., Gaynor J.W., Bach C.C., Hjortdal V.E., Østergaard J.R. (2016). Congenital Heart Defects and Indices of Placental and Fetal Growth in a Nationwide Study of 924 422 Liveborn Infants. Circulation.

[39] Leon R.L., Sharma K., Mir I.N., Herrera C.L., Brown S.L., Spong C.Y., Chalak L.F. (2022). Placental Vascular Malperfusion Lesions in Fetal Congenital Heart Disease. Am. J. Obstet. Gynecol..

[40] O’Hare C.B., Mangin-Heimos K.S., Gu H., Edmunds M., Bebbington M., Lee C.K., He M., Ortinau C.M. (2023). Placental Delayed Villous Maturation is Associated with Fetal Congenital Heart Disease. Am. J. Obstet. Gynecol..

[41] Ernst L.M. (2018). Maternal Vascular Malperfusion of the Placental Bed. J. Pathol. Microbiol. Immunol..

[42] Wright E., Audette M.C., Xiang Y.Y., Keating S., Hoffman B., Lye S.J., Shah P.S. (2017). Maternal Vascular Malperfusion and Adverse Perinatal Outcomes in Low-Risk Nulliparous Women. Obstet. Gynecol..

[43] Josowitz R., Linn R., Rychik J. (2023). The Placenta in Congenital Heart Disease: Form, Function and Outcomes. Neoreviews.

[44] Burton G.J., Jauniaux E., Burton G.J. (2021). Placentation in the human and higher primates. Placentation in Mammals.

[45] Gagnon R. (2003). Placental Insufficiency and Its Consequences. Eur. J. Obstet. Gynecol. Reprod. Biol..

[46] Neerhof M.G., Thaete L.G. (2008). The Fetal Response to Chronic Placental Insufficiency. Semin. Perinatol..

[47] Volpe J.J. (2009). Brain injury in premature infants: A complex amalgam of destructive and developmental disturbances. Lancet Neurol..

[48] Peyvandi S., Rollins C. (2023). Fetal Brain Development in Congenital Heart Disease. Can. J. Cardiol..

[49] Matthews J., Rajakumar B., Carreon C.K., Morton S.U. (2024). Placental—Heart Axis: An Evolutionary Perspective. Int. J. Mol. Sci..

[50] Schlatterer S.D., Murnick J., Jacobs M., White L., Donofrio M.T., Limperopoulos C. (2019). Placental Pathology and Neuroimaging Correlates in Neonates with Congenital Heart Disease. Sci. Rep..

[51] Radford B.N., Zhao X., Glazer T., Eaton M., Blackwell D., Mohammad S., Lo Vercio L.D., Devine J., Shalom-Barak T., Hallgrimsson B. (2023). Defects in placental syncytiotrophoblast cells are a common cause of developmental heart disease. Nat. Commun..

[52] Wilson R.L., Yuan V., Courtney J.A., Tipler A., Cnota J.F., Jones H.N. (2022). Analysis of commonly expressed genes between first trimester fetal heart and placenta cell types in the context of congenital heart disease. Sci. Rep..

