# Supplementary material for: Diminished Late Gestation Placental Volume in Fetal Heart Disease and Implications for Birth Anthropometrics
Source: J Cardiovasc Dev Dis. 2026 May 31;13(6):236. doi: 10.3390/jcdd13060236 (PMC13301887; doi:10.3390/jcdd13060236)
Supplement: Supplementary file 1 [file jcdd-13-00236-s001.zip › jcdd-4318106-supplementary.pdf]

Supplemental Material

Supplemental Figure 1. Placental MRI Images

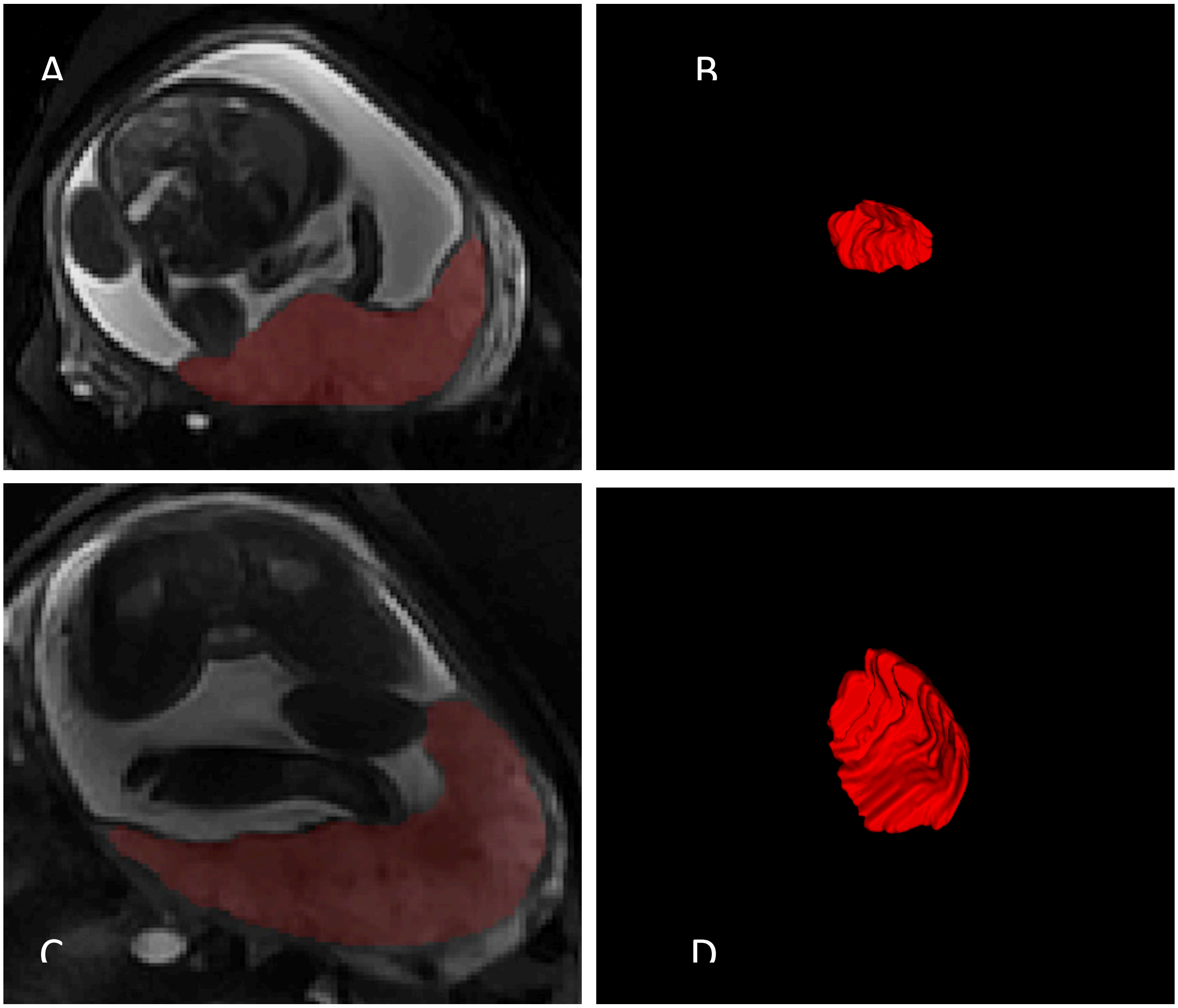

Supplemental Figure 1: Magnetic resonance image of an in-vivo placenta with segmentation mask and 3-D reconstruction in a fetus with CHD at 25-weeks gestation (A-B) and 32-weeks gestation (C-D)

Supplemental Table 1. Raw Placental Volume MRI Data from Control Subjects Utilized to Create PV Z-Scores

GA Range	*PV Mean*	*PV SD*	*Count*
16-17	261.0611	40.99965	2
17-18*	NA	NA	NA
18-19	223.6004	22.17514	4
19-20	220.3339	81.71972	6
20-21	324.3246	95.53091	7
21-22	393.1468	41.01015	3
22-23	330.1278	78.17561	5
23-24	429.9096	156.74269	5
24-25	417.3368	101.81846	6
25-26	482.2129	83.32442	17
26-27	446.8750	118.60130	21
27-28	470.9705	83.17842	14
28-29	519.4630	90.11030	20
29-30	660.1004	111.41719	7
30-31	528.2581	190.65065	10
31-32	748.3714	162.61727	10
32-33	750.8773	155.86691	12
33-34	783.1063	169.52026	14
34-35	803.2725	142.84146	20
35-36	752.3884	186.22133	19
36-37	844.0907	181.82436	26
37-38	858.7945	142.83726	17
38-39	818.2665	163.84632	11
39-40	1014.7703	148.70714	5
*No control or CHD scans in this GA range			

Supplemental Figure 2. Cohort

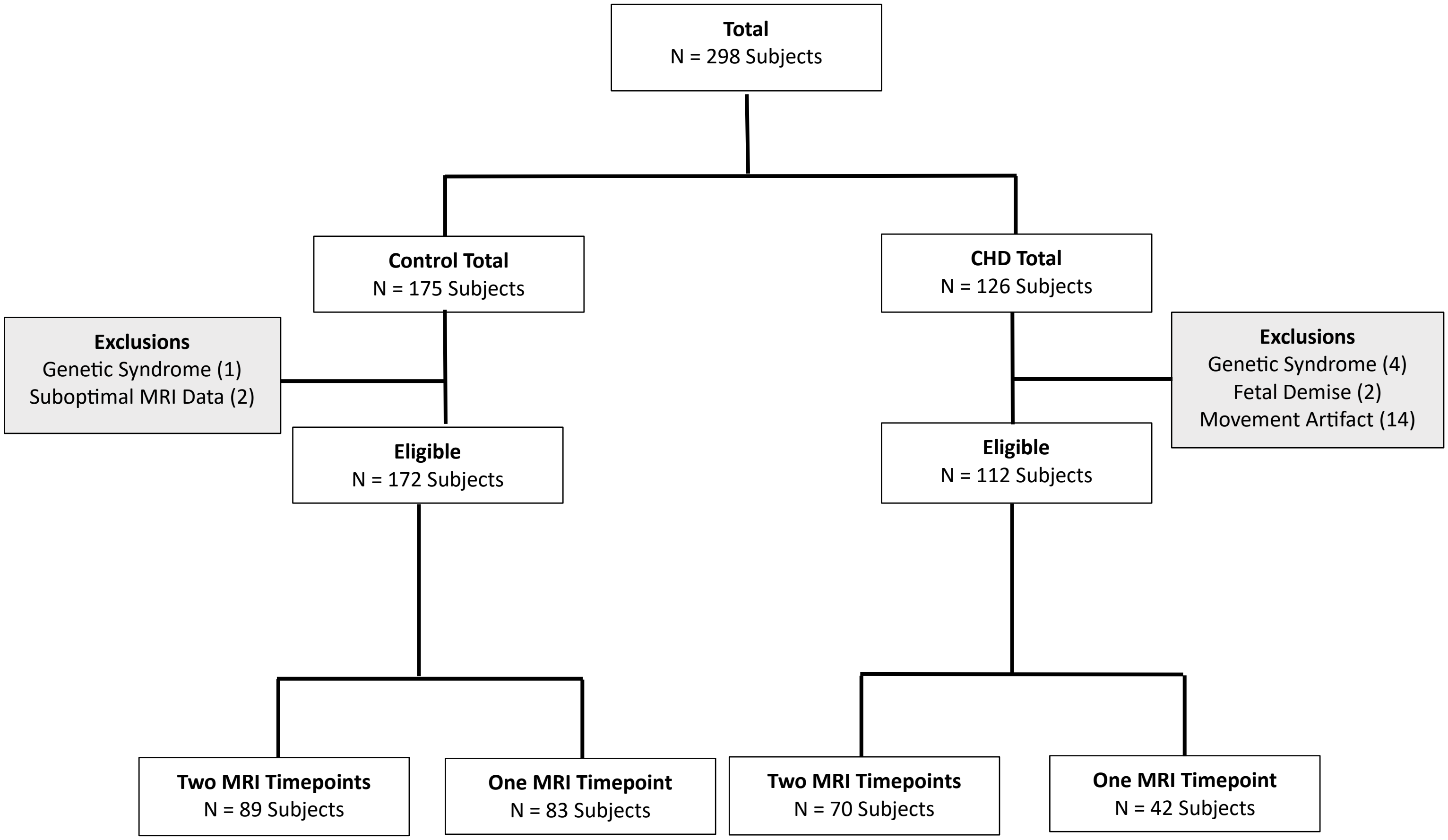

Supplemental Table 2. Demographics of CHD Cohort

Variable<br>*N (%); Mean ± SD*	*CHD*<br>*N = 112*
Sex of Fetus – male	74 (66)
Primary CHD Class	
Class 0 (TGA)	25 (22)
Class I (excluding TGA)	30 (27)
Class II	14 (13)
Class III	15 (13)
Class IV	28 (25)
CHD Lesion	
dTGA (with or without IVS/VSD)	26 (23)
HLHS	20 (18)
Other functional single ventricle anomaly	20 (18)
Other	15 (13)
TOF (With or without PA or MAPCAs)	13 (12)
VSD (with or without IAA/Coarctation)	6 (5)
Truncus Arteriosus	5 (4)
Coarctation/Aortic Arch Hypoplasia	2 (2)
PA/IVS	2 (2)
TAPVC	1 (0.9)
Fertility Methods Used for Pregnancy – Yes	7 (6)
Unknown	8 (7)
Maternal Medical History	
Diabetes	9 (8)
Hypothyroidism	5 (4)
Hyperthyroidism	0 (0)
Epilepsy	0 (0)
Pregnancy Induced Hypertension	2 (2)
Prenatal Exposures	
Alcohol	2 (2)
Cigarettes	4 (4)
Marijuana	3 (3)
Unknown	9 (8)
Prenatal Genetic Testing	
Yes	57 (51)
Unknown	15 (13)
Mode of Delivery	
Cesarean Section (elective)	27 (24)
Cesarean Section (emergency)	4 (4)
Cesarean Section (unknown cause)	15 (13)
Vaginal	57 (51)
Unknown	9 (8)
Gestational Age at Birth (weeks)	38.61 ± 1.14
Birth Length (in)	49.30 ± 3.00
Birth Head Circumference (cm)	33.29 ± 1.87

CHD: congenital heart disease; HLHS: hypoplastic left heart syndrome; dTGA: d-transposition of the great arteries; IVS: intact ventricular septum; TOF: Tetralogy of Fallot; PA: pulmonary atresia; MAPCAs: major aorta-pulmonary collateral arteries; VSD: ventricular septal defect; IAA: interrupted aortic arch

Supplemental Table 3. Demographics of CHD Cohort: 1 vs. 2 MRI studies

Variable	One MRI	Two MRIs	p-value
Maternal Age at First MRI	34 (31-37)	34 (30-36)	0.57
GA at First MRI	32.9 (29.5-34.6)	30.8 (28.4-34.3)	0.13
GA at Birth	39 (38.7-39.4)	39 (38.1-39.4)	0.44
Median (IQR); p-value calculated with Wilcoxon rank-sum test			

Supplemental Figure 3. CHD vs Control Raw Placental Volumes Across Gestation

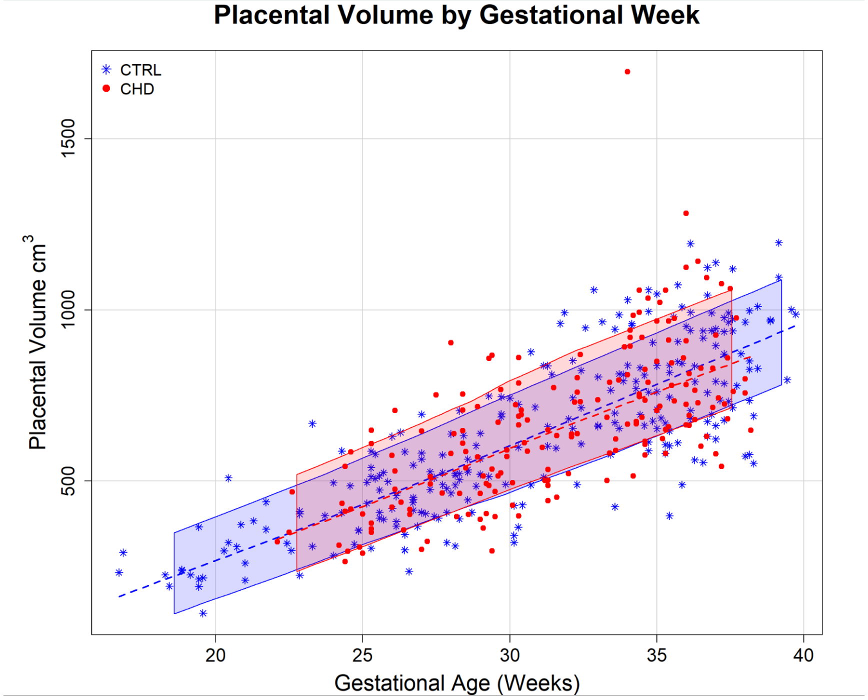

Supplemental Table 4. Linear Mixed Effects Model of Association of Placental Volume in CHD vs CTL

Analysis	Factors	Estimate	SE	T-value	P-value
**Unadjusted**	CHD vs. CTL	-20.29	24.56	-0.826	0.409
**GA Adjusted**	CHD vs. CTL	-12.57	21.36	-0.588	0.557
	Gestational Age at Scan	35.89	1.11	32.24	<0.001
**GA & Demographics Adjusted**	CHD vs. CTL	-1.17	29.23	-0.04	0.968
	Gestational Age at Scan	36.15	1.36	26.54	<0.001
	Fetal Sex (Male vs. Female)	-1.92	25.92	-0.07	0.941
	Race (Black)	32.23	36.03	0.89	0.373
	Race (Hispanic)	74.18	42.15	1.76	0.081
	Race (Asian)	-35.24	58.61	-0.60	0.549
	Race (Other)	-55.92	47.89	-1.17	0.245
	Education (Secondary)	-39.69	42.14	-0.94	0.348
	Education (Graduate)	-11.84	49.28	-0.24	0.811
	Maternal Age at Scan	-1.95	2.49	-0.78	0.435

Linear Mixed Effects Model (LME); Race Reference = White; Education Reference = High School

Placental Volume (cm^3); CHD = Congenital Heart Defects; CTL = Control, GA = Gestational Age

Least Squares Means: CTL 671, CHD 670

Supplemental Table 5. Linear Mixed Effects Model Demonstrating the Association of CHD Cohort Placental Volume Z-Scores with CTL Population Mean

Analysis	Factors	Estimate	SE	T-value	P-value
**Unadjusted**	CHD vs. CTL	-0.14	0.16	-0.89	0.374
**GA Adjusted**	CHD vs. CTL	-0.14	0.16	-0.89	0.373
	Gestational Age at Scan	-0.0004	0.008	-0.047	0.962
**GA & Demographics Adjusted**	CHD vs. CTL	-0.08	0.20	-0.373	0.710
	Fetal Sex (Male vs. Female)	-0.10	0.18	-0.554	0.581
	Race (Black)	0.25	0.25	1.004	0.318
	Race (Hispanic)	0.44	0.29	1.485	0.141
	Race (Asian)	-0.32	0.41	-0.785	0.434
	Race (Other)	-0.32	0.34	-0.950	0.344
	Education (Secondary)	-0.27	0.29	-0.910	0.365
	Education (Graduate)	-0.09	0.34	-0.249	0.804
	Maternal Age at Scan	-0.01	0.02	-0.458	0.648

Linear Mixed Effects Model (LME); Race Reference = White; Education Reference = High School Placental Volume (cm^3); CHD = Congenital Heart Defects; CTL = Control, GA = Gestational Age
